# The Influence of Individual and Team Mindfulness on Work Engagement

**DOI:** 10.3389/fpsyg.2019.02928

**Published:** 2020-01-21

**Authors:** Shengmin Liu, Huanhuan Xin, Li Shen, Jianjia He, Jingfang Liu

**Affiliations:** ^1^School of Management, University of Shanghai for Science and Technology, Shanghai, China; ^2^School of Management, Shanghai University, Shanghai, China

**Keywords:** individual mindfulness, team mindfulness, recovery level, work engagement, conservation of resources

## Abstract

Mindfulness metacognitive practice that can be performed in the workplace. Drawing on the theory of conservation of resources, we test a moderated mediating model of how and when employee mindfulness has a positive effect on work engagement. Via analysis of data from 311 employees from 83 teams at different times, this study investigates the relationship between employee mindfulness and work engagement as well as the moderating effect of team mindfulness and the mediating effect of recovery level. The results from this multi-wave field study show that the mindfulness of the individual employee has a positive influence on work engagement and that recovery level plays a mediating role. Team mindfulness positively moderates the relationship between individual mindfulness and work engagement. This conclusion may bridge the relationship between mindfulness and work engagement theory.

## Introduction

An increasing number of researchers and managers are now focusing on how to deal with the anxiety and distraction of employees and improving their level of well-being and work engagement. Mindfulness is receiving growing attention from scholars and organizational managers as a means of solving these problems. Mindfulness means attention to the present, with awareness and non-judgmental processing (Kabat-Zinn, 2005). When mindfulness is applied in many fields such as psychology, neuroscience, and medicine, it can help individuals to keep calm, maintain mental sobriety, and achieve a better level of attention (Wadlinger and Isaacowitz, 2011). Research in the field of organizational management has shown that mindfulness helps individuals pay more stable and effective attention to information related to current tasks (Dane, 2011).

Work engagement is defined as “a positive, fulfilling work-related mental state characterized by vitality, dedication and concentration” (Schaufeli et al., 2002). Recently, work engagement has become one of the most important concepts in the field of management. Work engagement plays a positive influence on employee performance (Rich et al., 2010), and recovery also shows positive effects on work engagement (Sonnentag, 2003). Recent studies have found that trait mindfulness and mindfulness training are positively correlated with work engagement (Leroy et al., 2013; Malinowski and Lim, 2015). However, the current state of knowledge on the relationship between mindfulness and work engagement remains very limited. A few studies have shown that specific functions, positive emotion, and psychological capital help to build an important path through which mindfulness can influence work engagement (Leroy et al., 2013; Malinowski and Lim, 2015). However, these studies mainly explored the functional mechanism of mindfulness from the perspectives of the individual’s own feelings or their characteristics. Our study will focus on how employee mindfulness affects work engagement at the individual and team levels.

According to the field dynamic theory, individuals in an organization are embedded in the environment, and their individual attitudes and behavioral expressions are the interacting results of their own characteristics (including motivation, personality, etc.) and contextual factors (including team characteristics, interpersonal relationships, etc.) (Carsten et al., 2010). As a result, contextual factors may provide a new insight into how mindfulness affects work engagement. Based on the field dynamic theory, testing the moderated mediating model between employee mindfulness and work engagement has important theoretical implications. Mindfulness brings better consciousness and regulation to individuals (Brown and Ryan, 2003) so that they can recover from stress as quickly as possible. Meanwhile, team characteristics, as the important contextual factors, can moderate the relationship between individual mindfulness and work engagement. Team mindfulness, as one of the most important team characteristics, has a certain influence on employees’ attitudes and behavior. It is appropriate to use team mindfulness as a moderator of the mechanism between individual mindfulness and work engagement. This study explores the mechanism that governs whether mindfulness will influence employees’ work engagement via the recovery level and tests the moderating role of team mindfulness between individual mindfulness and recovery level.

### Theory and Hypotheses

The concept of mindfulness can initially be traced back to the process of Buddhist practice, and early scholars considered mindfulness as a state of attention to the present; Thera (1970) proposed that mindfulness is a clear and accurate understanding of what is perceived in the heart in a changing sensory world. Since the start of the present century, mindfulness research in clinical practice has grown rapidly (Lao et al., 2016). When mindfulness was applied to the field of medicine, researchers found that mindfulness had a regulatory and helpful effect on physical and mental health, and the application of mindfulness was then gradually extended to the sports industry for team cooperation. Nowadays, mindfulness is well-accepted in the field of management research, where it is defined as “an acceptable attention and awareness of current events and experiences” (Brown et al., 2007). Mindfulness here can refer to either an individual’s state of consciousness or an individual trait (Brown and Ryan, 2003). Trait mindfulness is an inherent trait within an individual. Based on personal traits, the relatively stable and important factors of work engagement, this study focuses on the impact of the team’s subordinate trait mindfulness on work engagement.

Mindfulness provides teams and employees with a high level of attention to the quality of the moment, and previous studies have found that this particular quality significantly improves the physical and mental health of behavioral subjects (Jamieson and Tuckey, 2017). Thus, the recovery level will be used as an intermediary variable through which mindfulness influences work engagement in this study.

### Individual Mindfulness and Work Engagement

According to conservation of resources (CORs) theory, the acquisition of initial resources will bring more resources to individuals, and a scarcity of resources will lead to further depletion of resources in future (Hobfoll, 2001). When people have a greater resource endowment, it will be easier for them to obtain resource benefits. Conversely, individuals with fewer resources are more likely to suffer resource losses. According to COR theory, employee mindfulness, as a special internal resource of an individual, helps to enhance the positive psychological resource of work engagement. Attention is one of the core elements of mindfulness (Good et al., 2016). When employees can maintain active observation and attention to the current internal and external stimuli, they can maintain a high degree of vigilance and sensitivity to their own internal experience and external situation in their daily work. That is, employees with high mindfulness are more likely to maintain a beginner’s mind, concentrate their attention on current activities, regard each moment as unique, and finally experience a higher level of vitality. Meanwhile, employees with high mindfulness can be free from the interference of various unnecessary factors because they are in a “focus on the present” state. By actively manipulating and controlling attention, people can focus their attention on tasks and then devote themselves wholeheartedly to their work. Because of their focus on current experiences and events, highly mindful employees can evaluate the effects of habitual, automated, and unconscious understanding on current events, thereby avoiding the tendency to put a subjective bias on perception and processing (Brown and Ryan, 2003). As a result, highly mindful employees are less likely to engage in impulsive behavior, even when they meet difficulties. This helps them to maintain a state of balance and to avoid being negatively affected by the outside environment (Brown and Ryan, 2003) so that they can retain more time and energy to return to work and immerse themselves in the work. Besides, as an important internal resource, mindfulness enables the employees to focus on their inner experiences and outside things in a non-judgmental way (Dane, 2011). As a result, highly mindful employees tend not to view obstructive events as challenging or demanding, and neither do they subjectively evaluate negative situations. This helps the employees to eliminate distractions from the past and negative emotional experiences, which enables them to finish their tasks energetically with a positive, optimistic attitude. Based on the CORs theory, an individual with high mindfulness can get more internal psychological resources, such as high attention, objective evaluation, and concentration on work. Hence, it can be expected that employees with high mindfulness can obtain positive psychological resources such as job involvement by adjusting and controlling their own cognition, attention, and emotion (Good et al., 2016).

Existing research has found that mindfulness has a positive effect on work engagement. For example, Leroy et al. (2013) apply self-determination theory to predict that individual mindfulness can influence work engagement through two paths, that is, directly by making people more attentive and focused and indirectly by improving people’s inner awareness, resulting in a higher level of authenticity function. According to the above discussion and combined with the characteristics of employee mindfulness in the workplace, mindfulness may influence work engagement in three ways. ① Stable attention. Achieving a sustained and stable focus on the present is a central feature of mindfulness. Individual mindfulness reduces the habit of roaming thoughts and attention through steady attention because it improves attention efficiency and is more likely to keep the individual in a state of concentration (Wadlinger and Isaacowitz, 2011). At the same time, the individual is aware of the clarity and vividness of the experience because of their state of receptive attention, so the individual is immersed in happiness and will become more proactive in participating in activities (Brown et al., 2007). ② Self-awareness. Brown et al. (2007) believe that mindfulness supports work by enhancing self-awareness of emotions, thoughts, and behaviors, supporting action based on one’s own core or true self-consciousness, and fostering more autonomous motivation. This motivation not only encourages employees to actively acquire and obtain the resources they need but also prompts them to put more energy and enthusiasm into work tasks. ③ Self-regulation. Mindfulness can help people experience and achieve more current attention and awareness (Glomb et al., 2011). Specifically, mindfulness can help individuals better manage themselves and reduce autonomous behavior (Glomb et al., 2011). As a result, the following assumption is made:

H1: Individual mindfulness is positively correlated with work engagement.

### The Mediating Role of Recovery Level

According to CORs theory, individuals tend to prevent their losses or acquire new resources (Halbesleben et al., 2014). Employees with high mindfulness may avoid further loss of their resources. They can also control the negative emotions caused by stress. Unlike mindfulness, all self-controlling behaviors require inhibition, leading to limited resources (Johnson et al., 2017). Suppressing attention to many stressors would lead to further resource loss for the employees (Hobfoll, 2001). The key requirement of mindfulness is acceptance of the present focus and experience, avoiding stressors from the past or the future (Brown and Ryan, 2003). It implies that individuals with high mindfulness can accept the present and even negative events instead of deliberately suppressing negative emotions and psychological reactions. Recovery level is the degree to which an individual can recover from stress or boredom. By experiencing their negative emotions with no control, employees with high mindfulness can release process-inhibiting emotions, avoid expending psychological energy, and conserve their current recovery level. In addition, with non-judgment and focus on the present moment, individuals may acquire psychological benefits by experiencing greater autonomy. Experiencing control can replenish individuals by satisfying the basic need for autonomy (Ryan and Deci, 2000). Previous research supports the positive effect of control awareness on recovery level (Sonnentag and Fritz, 2007).

A high level of recovery may promote the process of work engagement or improve investment of cognitive and emotional energies in the workplace (Kahn, 1990; Schaufeli et al., 2002; Rich et al., 2010). Employees with high engagement contribute a high level of energy and attention to their tasks (Schaufeli et al., 2002; Sonnentag et al., 2010), enrich their well-being, and improve their job performance (Bakker et al., 2008; Christian et al., 2011). The recovery level shows the individual’s ability to eliminate negative events and increase positive events. Employees can get new resources from other activities, such as reading books, learning new skills, and doing exercise. Employees with a high recovery level will relax and gain self-efficacy easily, so that they have more energy for work-related tasks and tend to make more effort (Hahn et al., 2011). According to CORs theory, individuals tend to search for new resources to satisfy their needs. If employees cannot recover from work stress and fatigue, they will lose more resources. That is to say, employees will have to get new resources for recovery. The employees’ interpersonal relationship is more resilient when they are confronted with conflict or extreme events, and they can more easily and energetically pay attention to the task and exclude irrelevant effects from the workplace (Kahn, 1990; Sonnentag, 2003). Contrarily, employees with insufficient recovery lack the resources to expend high effort, deal with stress, or concentrate on tasks (Sonnentag, 2003). Previous studies have proved this positive impact of recovery level on work engagement (Sonnentag et al., 2010; Qin et al., 2018). Because individual mindfulness helps employees to improve their recovery level, which is a proximal antecedent of work engagement, we propose that the recovery level plays a mediating role in the relationship between individual mindfulness and engagement.

H2: Individual mindfulness is positively correlated with work engagement via recovery level.

### The Moderating Role of Team Mindfulness

Individual mindfulness is beneficial for employees according to CORs theory. Individual and situational factors can affect the reactions of preventing loss and acquiring new resources (Hobfoll et al., 1990; Halbesleben et al., 2014). Especially, the individual can replenish their energy after events (Halbesleben et al., 2014), which might have impacts on the resource-related consequences of individual mindfulness. Gunasekara and Zheng (2019) proved that surface acting can negatively moderate the relationship between individual mindfulness and work engagement because individuals expend more psychological resources when they are involved in surface acting. Furthermore, Zhang et al. (2018) found that leader mindfulness can positively moderate the relationship between individual mindfulness and work engagement.

Work states might influence the response model of how employees react to work stress. One work state that is highly correlated with individual mindfulness is team mindfulness, defined as “a shared belief among team members that team interactions are characterized by awareness and attention to present events and by experiential, non-judgmental processing of within-team experiences” (Yu and Zellmer-Bruhn, 2018). Individual mindfulness shows the cognition at the individual level, and team mindfulness is the mutual belief of team members at the collective level. Team mindfulness studies the whole team’s attention to the current experience as well as stimuli that are internal and external to the team. Considering the lower distraction from task actions (Good et al., 2016) in high team mindfulness, members can perceive greater nuance in the team (Morrison et al., 2014), preventing resource-consuming processes arising from irrelevant stimuli such as conflict expression (Slagter et al., 2011), so team mindfulness promotes the link from individual mindfulness to recovery level. Furthermore, mindful teams consider stress in more non-judgmental ways (Good et al., 2016), cutting negative reactivity (Glomb et al., 2011). The team members are unlikely to be affected by the conflict in this team atmosphere (Amason and Sapienza, 1997). The team’s awareness, as a team member’s shared belief, also affects the way the team members communicate and work. In addition, although individual mindfulness and team mindfulness belong to different levels of construct, the resonance between them can have the interacting impacts on recovery level because they have the same contents, such as non-judgmental processing and being open to the present, which both conserve resources for work engagement. Team mindfulness can influence the team operation process and help individuals deal with things and teammates more objectively. Employees in a mindful team are encouraged to communicate and get new information and resources actively. Under the influence of team mindfulness, individual mindfulness help individuals focus more on current events and not waste resources on negative events and emotions. As discussed above, employees must retain their own resources and acquire new resources in order to recover from work stress. Team mindfulness provides a relaxed and objective atmosphere that encourages employees to acquire new knowledge, energy, and methods. These new resources in a mindful team will naturally help improve the recovery level of team members. The higher the team mindfulness, the stronger the effect of individual mindfulness on recovery level.

H3: Team mindfulness moderates the positive indirect effect of individual mindfulness on work engagement via recovery level, such that the strength of this indirect effect is positively correlated to team mindfulness.

## Materials and Methods

### Sample Sources and Procedures

This study chose three service companies in Eastern China as its research sources. The study was supported by the senior management teams of these companies. In order to test the causal relationship, the data were collected at three times, and 350 members from 89 teams were invited to participate in the survey. At time 1, the research team required employees to fill in a survey about their scores for trait mindfulness, recovery level, and work engagement. Meanwhile, demographic information such as gender, age, and length of working day was provided by the human resources department of the surveyed enterprises. Three months later, at time 2, a second questionnaire was collected. The employees who had participated in the time 1 questionnaire were asked to report their team mindfulness and recovery level. Three months later, at time 3, a third questionnaire was collected, and the employees who had participated in the time 2 questionnaire were asked to fill in their work engagement scores. Finally, the data of 311 employees from 83 teams were obtained. In these samples, the proportion of women was 42%, the average age was 25.13 (*SD*: 5.06), and the average level of education was 2.04 (*SD*: 0.69).

### Variable Measurement

The measurement scales in this study are from Western scholars, so a translation-back translation procedure is used to ensure that the content of these items conforms to the author’s original intention (Brislin, 1990).

#### Individual Mindfulness (T1)

We measured this by the 15-item Mindfulness Attention Awareness Scale (Brown and Ryan, 2003). This scale is widely used to measure trait mindfulness. The items included: “I completed some of the activities in a hurry, but I did not really notice them.” The Cronbach’s alpha value was 0.89.

#### Team Mindfulness (T2)

We measured this with Yu and Zellmer-Bruhn’s (2018) 10-item Team Mindfulness Scale (α = 0.90), which includes items such as: “this team is friendly to members when things go wrong.” The mean Rwg was 0.81; ICC (1) was 0.17, and ICC (2) is 0.49, which indicated appropriate aggregation of teammate mindfulness.

#### Recovery Level (T1 and T2)

We measured this the Recovery Experience Questionnaire created by Sonnentag (2003). This is a three-item scale that is used to quantify recovery level. For example: “I feel relaxed.” The Cronbach’s alpha value was 0.79.

#### Work Engagement (T1 and T3)

We used the Utrecht Work Engagement Scale to measure work engagement. This is a three-item scale created by Schaufeli et al. (2006). A sample item is: “I am immersed in my work.” The Cronbach’s alpha value was 0.88.

The participants reported their level for all scales on a five-point Likert scale (1 = “Strongly disagree,” 5 = “strongly agree”).

### Analytic Strategy

Firstly, this study preliminarily verified the correlation between variables through a correlation test. Then, using confirmatory factor analysis (CFA), it examined the discrimination validity of key variables in the research model, including individual mindfulness, team mindfulness, recovery level, and work engagement. Given that the data at the individual level was nested in the team, the hypothesis test was conducted in this study using the multi-level structural equation modeling in Mplus 7.0. In addition, in order to isolate the cross-level influence from inter-group team interaction and avoid detection of false cross-level effects (David and Mark, 1998), in data processing, except for gender variables, all variables at the individual level (level 1) were centralized by group mean and all variables at the team level (level 2) were centralized by total mean. Finally, this study tested the mediation effect (hypothesis 2). Then, the Monte Carlo method was adopted to analyze the moderated mediating effect (hypothesis 3), which was the mediating effect of recovery level at different levels of team mindfulness (above or below one standard deviation). We controlled by the initial recovery level and work engagement at time 1 after the correlation between demographic variables and key variables was found to be insignificant.

## Results

### Correlation Analysis and CFA

The mean values, standard deviations, and correlation coefficients are shown in Table 1. Table 1 indicates that individual mindfulness was positively related with team mindfulness (*r* = 0.21, *p* < 0.05), individual mindfulness was positively related with work engagement (*r* = 0.41, *p* < 0.001), individual mindfulness was positively related with recovery level (*r* = 0.34, *p* < 0.001), and recovery level was positively related with work engagement (*r* = 0.27, *p* < 0.01).

**TABLE 1:** Descriptive statistics and correlations.

**Variable**	***M***	***SD***	**1**	**2**	**3**	**4**	**5**	**6**	**7**	**8**
(1) Sex	0.42	0.31								
(2) Age	25.13	5.06	0.03							
(3) Education	2.04	0.69	–0.03	0.07						
(4) Individual mindfulness	4.24	0.59	0.01	–0.02	–0.08					
(5) Recovery level	4.13	0.35	0.01	0.05	–0.02	0.34^∗∗∗^				
(6) Work engagement	3.54	0.61	–0.02	0.05	0.03	0.41^∗∗∗^	0.27^∗∗^			
(7) Team mindfulness	4.21	0.69	0.02	0.03	0.01	0.21^∗^	0.28^∗∗^	0.24^∗∗^		
(8) Initial recovery level	3.42	0.43	0.02	0.03	0.07	0.28^∗∗^	0.36^∗∗∗^	0.15	0.17	
(9) Initial work engagement	3.01	0.53	0.02	0.03	0.06	0.15	0.10	0.29^∗∗^	0.12	0.20^∗^

*For the individual level, *n* = 311, for the group level, *N* = 83, ^∗∗∗^*p* < 0.001, ^∗∗^*p* < 0.01, ^∗^*p* < 0.05, two-tailed. Team mindfulness uses the score from individual perception of the team before aggregation.*

The CFA results indicated that a four-factor model (individual mindfulness, recovery level, team mindfulness, and work engagement) achieved a more ideal state (χ^2^ = 721.43, *df* = 428, CFI = 0.97, RMSEA = 0.05) than a three-factor model combining recovery level and work engagement (Δχ^2^ = 332.92, Δ*df* = 3, CFI = 0.82, RMSEA = 0.12) and other models. As a result, the four-factor measurement model (individual mindfulness, team mindfulness, recovery level, and work engagement) had good discriminating validity.

### Hypothesis Test

**FIGURE 1:**
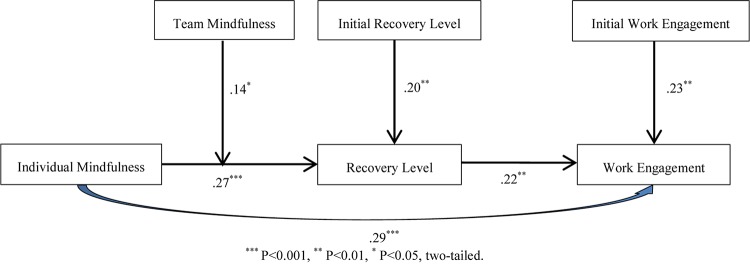
MSEM results for the theoretical model.

As shown in Figure 1, there was a significant positive relationship between individual mindfulness and work engagement after adding control variables (β = 0.29, *SE* = 0.07, *p* < 0.001), supporting hypothesis 1.

Aiming at testing the mediating effect, individual mindfulness was significantly correlated with recovery level (β = 0.27, *SE* = 0.09, *p* < 0.001), and recovery level was significantly positively correlated with work engagement (β = 0.22, *SE* = 0.08, *p* < 0.01), supporting hypothesis 2.

**FIGURE 2:**
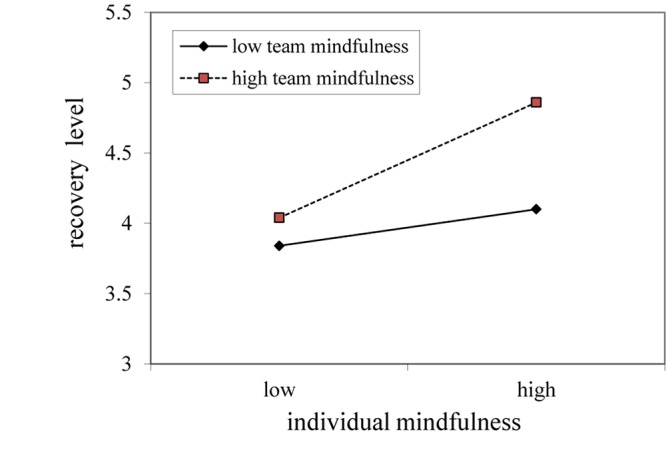
Moderation plots.

The interacting effect of team mindfulness and individual mindfulness on recovery level was tested; the results are shown in Figures 1, 2. The moderating role of team mindfulness on the relationship between individual mindfulness and recovery level was significantly positive (β = 0.14, *SE* = 0.07, *p* < 0.05). To further verify hypothesis 3, as shown in Figure 2, when team mindfulness was high (M + SD), the line showing the relationship between individual mindfulness and recovery level was steeper than that showing the relationship when team mindfulness was low (M-SD), partly supporting hypothesis 3.

Table 2 shows the moderated mediating role of team mindfulness. The results from the Monte Carlo method showed the moderated role of team mindfulness on the indirect effect of recovery level between individual mindfulness and work engagement. When team mindfulness was high (M + SD), the indirect effect was significant (γ = 0.15, 95%CI = [0.05, 0.19]); when team mindfulness was low (M-SD), the indirect effect was not significant (γ = 0.05, 95%CI = [−0.01, 0.11]). Meanwhile, the differentiation between high and low was significant (γ = 0.10, 95%CI = [0.07, 0.17]), supporting hypothesis 3.

**TABLE 2:** Indirect effects of individual mindfulness on work engagement (via recovery level) at low and high levels of team mindfulness.

	**Recovery**	**Work**	
**Individual mindfulness**	**level**	**engagement**	
Moderators	Indirect effect	LLCI	ULCI
Low team mindfulness (−SD)	0.05	−0.01	0.11
High team mindfulness (+ SD)	0.15	0.05	0.19
Differentiation (between −SD and + SD)	0.10	0.07	0.17

## Discussion

Based on CORs theory, this study reveals the process by which mindfulness helps employees to improve their work engagement through the mediating process of recovery level. The results show that there is a positive indirect relationship among individual mindfulness, recovery level, and work engagement. The results also show that team mindfulness plays a moderated role in helping employees recover from work stress. These results further reveal the mechanism by which mindfulness affects work engagement, and this study has some theoretical contributions and practical value.

### Implications for Theory

This study further explores the effects of mindfulness on employee work behaviors and attitudes. Although some scholars have previously studied the effect of mindfulness on employees’ work engagement, we study this mechanism with recovery level. This provides a new perspective on how mindfulness influences work engagement. Specifically, mindfulness help individuals to accept and focus on the present in order to recover from work stress. Therefore, employees can adjust their emotions and psychology and return to their original state of mind, and they can engage in the workplace.

The research findings are helpful for understanding the effect of mindfulness from the interactive perspective of the team and individual levels, and also increase the predictive effect of theory. Previous studies have focused on the effects of individual mindfulness on individual behavior and attitudes. Individuals work in teams, and their behaviors are bound to be influenced by team factors. This study explores both individual mindfulness and team mindfulness, which allow us to understand the concept and function of mindfulness from different research levels better. We creatively integrate group mindfulness into the influencing mechanism of individual mindfulness and work engagement. On the individual level, mindfulness encourages employees to focus more on their work by affecting their own attention and awareness. On the team level, team mindfulness is a construct above the individual level, which can moderate the behavior of team members. The role of team mindfulness is explored from the moderated boundary. Team mindfulness is different from individual mindfulness, but it can help individual mindfulness to maintain psychological resources. As a result, this conclusion makes the model more complete and responsive to the boundaries of positive effects in mindfulness. We adopt a new perspective for future research on mindfulness. At the same time, the conclusion also supports previous research, such as that showing the promotion of mental health in mindfulness. In short, the study explains that the team and individual mindfulness have different effects on employee behavior and thus further expands the breadth of the research subjects. It also supports results showing mindfulness to be a kind of trait that contributes to better work engagement and why and when mindfulness influences work engagement. The intermediary mechanism is unearthed from the aspect of recovery level.

From the perspective of resource conservation, this study analyses the mechanism operating between individual mindfulness and work engagement. The CORs theory emphasizes that individuals will take the initiative to acquire resources and protect the resources they originally possessed to meet their own needs. Firstly, the research results support the viewpoint of this theory. Individual mindfulness makes employees accept and not waste additional resources to deal with negative things. Individuals need resources to achieve a good recovery level. Recovery saves more energy and resources, allowing employees to pay more attention to their work. The acquisition and preservation of resources contributes to the establishment of this mechanism.

### Implications for Practice

The research has practical significance. Firstly, work engagement has a positive effect on employee performance. More and more research works are focusing on the antecedents of work engagement. Our research reveals some antecedents of work engagement, and provide a reference for enterprises to effectively promote employees’ work performance.

Secondly, in view of the positive effect of individual mindfulness in helping to restore and improve work engagement, mature mindfulness training programs should be adopted in organizational training and development to improve employee mindfulness. Currently, the most popular mindfulness training methods are mindfulness stress reduction and mindfulness cognitive therapy. The effective period of these training methods is relatively long. Thus, enterprises can bring the short training methods of valuable mindfulness training to their employees according to their own situations. From the perspective of employees, they can consciously cultivate their own mindfulness, and improve their level of mindfulness through participating in formal or informal training so that they can recover psychologically more quickly. Finally, given the positive effect of team mindfulness on employee mindfulness, managers can actively promote team mindfulness through team development and collective mindfulness training. This will enable the team to finish tougher tasks involving more conflicts or challenges. After mindfulness training, the team can use special time to meditate and think, to strengthen their level of mindfulness, and to help individuals promote resource recovery.

### Limitations and Future Research

First, this study uses a single of source data from employees and a causal relationship design, but single-source data shows common method biases (Podsakoff et al., 2003). The results of the self-reported data may deviate from the actual situation. Self-reported data are well-fitted to implicit constructs like mindfulness, recovery level, and work engagement (Berry et al., 2012), and we measured at three times to migrate the biases.

Second, given the boundary conditions of the mindfulness effect, there should also be negative conditions at the team level that inhibit the mediating effect of recovery level on the relationship between mindfulness and work engagement. Such negative boundary factors will be explored in the future. Third, the survey samples in this study are relatively similar. In order to increase external validity, different sample groups need to be selected to improve the universality of the conclusion. In addition, this paper studies the effect of trait mindfulness on work engagement. We suggest that to predict the effects of mindfulness interventions on employees in the future, it should be explored whether individuals who have received mindfulness training have improved recovery level and work engagement. This will enable mindfulness to be better applied in the workplace.

Finally, recovery requires the individual to accumulate mindfulness continuously in the natural scene. but this study only used cross-sectional data from three time points, which cannot fully reflect the continuous natural state of employees recovering from stress. Moreover, we only discussed the influence of mindfulness level on work engagement but did not research how the improvements of employee mindfulness and team mindfulness influence it. In the future, experiencing samples within the personal level can be used to explore the continuous influence of mindfulness on recovery level. Moreover, scholars can further verify the influence of mindfulness training on individual mindfulness level and how to make individuals accept mindfulness intervention.

## Data Availability Statement

The datasets analyzed in this article are not publicly available. Requests to access the datasets should be directed to lsm19801222@126.com.

## Ethics Statement

The studies involving human participants were reviewed and approved by the Interdisciplinary Committee on Ethics in Human Research (ICEHR). The patients/participants provided their written informed consent to participate in this study.

## Author Contributions

SL designed the moderated mediating model and wrote the literature review and hypotheses. HX wrote the data collection and analysis. LS wrote the introduction. JH wrote the results. JL wrote the discussion.

## Conflict of Interest

The authors declare that the research was conducted in the absence of any commercial or financial relationships that could be construed as a potential conflict of interest.
